# Advances in the Emerging Gradient Designs of Li Metal Hosts

**DOI:** 10.34133/2022/9846537

**Published:** 2022-08-01

**Authors:** Wanqing Guan, Xiaoqi Hu, Yuhang Liu, Jinmeng Sun, Chen He, Zhuzhu Du, Jingxuan Bi, Ke Wang, Wei Ai

**Affiliations:** ^1^Frontiers Science Center for Flexible Electronics (FSCFE), Xi'an Institute of Flexible Electronics (IFE) and Xi'an Institute of Biomedical Materials & Engineering (IBME), Northwestern Polytechnical University (NPU), 127 West Youyi Road, Xi'an 710072, China; ^2^School of Materials Science and Engineering, Nanyang Technological University, 50 Nanyang Avenue, Singapore, Singapore 639798

## Abstract

Developing host has been recognized a potential countermeasure to circumvent the intrinsic drawbacks of Li metal anode (LMA), such as uncontrolled dendrite growth, unstable solid electrolyte interface, and infinite volume fluctuations. To realize proper Li accommodation, particularly bottom-up deposition of Li metal, gradient designs of host materials including lithiophilicity and/or conductivity have attracted a great deal of attention in recent years. However, a critical and specialized review on this quickly evolving topic is still absent. In this review, we attempt to comprehensively summarize and update the related advances in guiding Li nucleation and deposition. First, the fundamentals regarding Li deposition are discussed, with particular attention to the gradient design principles of host materials. Correspondingly, the progress of creating different gradients in terms of lithiophilicity, conductivity, and their hybrid is systematically reviewed. Finally, future challenges and perspective on the gradient design of advanced hosts towards practical LMAs are provided, which would provide a useful guidance for future studies.

## 1. Introduction

The rapid development of electronics industry requires rechargeable batteries with higher energy density urgently [1–3]. Li metal anode (LMA) is one of the cutting-edge research topics by virtue of its high theoretical capacity (3860 mAh g^−1^) associated with the lowest electrode potential (-3.04 V *vs.* standard hydrogen electrode) [4–6]. When coupled with conversion cathodes, for example, sulfur, the energy density of the resulting Li-S battery reaches several times higher than current commercial Li-ion batteries [7, 8]. Therefore, Li metal batteries (LMBs) have been considered as one of the most promising candidates for the next-generation high-energy batteries [9–11]. Nevertheless, the practical application of LMBs is still impeded by the intrinsic drawbacks of LMAs [12–16]. On one hand, the low surface energy and migration energy of Li tend to form irregular Li plating/stripping, initiating rampant Li dendrite growth [17–23]. Once Li dendrites pierce the separator, they will trigger the internal short circuit and thermal runaway of LMBs, resulting in battery failure or even fire and explosion [24, 25]. On the other hand, because of the hyperactivity of Li, solid electrolyte interface (SEI) will be generated at the interface between metallic Li and electrolyte during the initial Li nucleation process [26–28]. The inherent SEI with rigid and fragile nature could be readily damaged by stress concentration upon cycling, which leads to “hot spots” for fast dendritic growth as well as undesired side reactions [29–32]. Moreover, the “hostless” feature of Li metal causes severe volume fluctuations during Li plating/stripping, which may further break the preexisted SEI layer and thus depletes the electrolyte and active Li metal significantly [33–38]. These issues inevitably bring premature failure or even safety concerns of LMBs [39].

**Figure 1:**
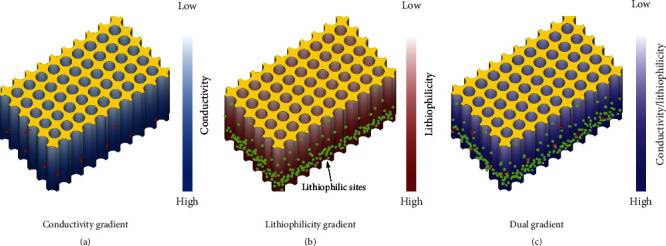
Schematic illustrations for the gradient designs of Li metal hosts: (a) conductivity gradient, (b) lithiophilicity gradient, and (c) dual gradient.

To date, great efforts in terms of exploring novel electrolytes [40, 41], artificial SEI [42, 43], modified separators [44], and Li metal hosts, [45–47] have been made to develop reliable LMAs [48]. Among them, introducing hosts shows great prospects, since it can not only accommodate the deposited Li but also inhibit the associated volume expansion [49, 50]. More importantly, according to the Sand's model, hosts with large specific surface area and rich porosity are conducive to reducing the local current density of LMAs; hence, it is beneficial for delaying dendritic Li formation [51]. Generally, the deposition patterns of Li in hosts can be classified into three modes, including top deposition, inner deposition, and bottom deposition [52–54]. In a typical top deposition process, Li metal prefers to deposit on the top of the preformed Li, which can easily initiate the generation of Li dendrites because of the “tip effects” and excessive local current density [55, 56]. In the case with nanostructured hosts introduction, a distinct Li deposition manner, namely, inner deposition, is noted, where the growth of Li dendrites can be suppressed to a certain extent. Nevertheless, Li still preferentially deposits on the upper surface under high current densities. To this end, bottom-up deposition, meaning Li grows gradually from bottom to upper layer, is believed to be an efficient pathway for retarding Li dendrite growth [57]. As a consequence, it is highly pursued to impart host materials with gradient properties for guiding Li deposition evenly. Since conductivity and lithiophilicity are two major factors that determine Li deposition/stripping processes [58], the gradient designs of host in terms of conductivity, lithiophilicity, and their hybrid are proposed [59–61]. As schematically illustrated in Figure 1(a), conductivity gradient represents hosts with stepwise declined conductivity from bottom to top side, which concentrates electrons at the bottom region and hence preferential Li deposition at the bottom of hosts. Likewise, lithiophilicity gradient suggests the gradient decreased lithiophilicity of the hosts from bottom to top (Figure 1(b)), which can guide Li^+^ to accumulate at the bottom for deposition. Correspondingly, dual gradient takes the advantages of conductivity and lithiophilicity designs (Figure 1(c)); thus, it is capable of regulating the distributions of electrons and Li^+^ simultaneously, which synergistically optimize the Li deposition behaviors of the hosts [12, 61]. Although gradient design of hosts demonstrates a huge potential in fabricating advanced LMAs [62–67], so far, a systematic and specialized overview of this interesting field is still absent. Herein, we endeavor to provide an in-depth overview on the emerging gradient designs of Li metal hosts by means of conductivity, lithiophilicity, and their hybrid for dendrite-free LMAs. The structural designs and underlying mechanisms of these gradient hosts for suppressing Li dendrite growth are discussed in detail. Besides, key scientific challenges and opportunities for further research are outlined as well. We believe this review would not only shed insights on the rational design of Li metal hosts but also stimulate more valuable investigations in this field.

## 2. Conductivity Gradient

Based on phase field simulations, Huang et al. revealed that the potential near host during Li plating is closely related to its conductivity [95]. In a high conductivity host, the uniform distribution of electric potential is able to uniformize Li^+^ flux and reduce the overpotential, which is beneficial for smoothing Li deposition [65]. Despite suppressed Li dendrite growth, the shorter Li^+^ diffusion path at the top can still result in Li deposition on the surface of the electrode, especially under the extreme conditions including high rate and/or high cycling capacity [9]. Consequently, constructing conductivity gradient that is capable of adjusting the surrounding potential distribution of host has been proved to be a feasible route for realizing bottom-up Li deposition. In general, conductivity gradient hosts can be divided into two categories, that is, free-standing hosts and integrated hosts. Free-standing hosts are frameworks with conductivity which gradually decreases from bottom to top, while integrated hosts are consisted by poor conductive frameworks covered on current collectors. As a result of conductivity differences along the vertical direction, more electrons are concentrated at the bottom region of the hosts for preferential Li deposition.

**Figure 2:**
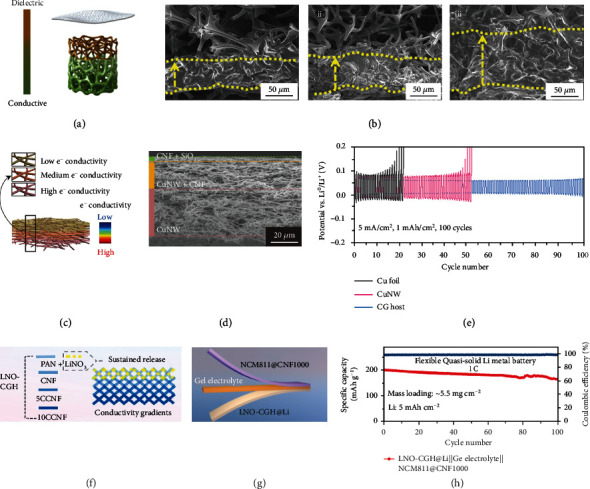
(a) Schematic illustration of the CDG-sponge. (b) Cross-section SEM images of the CDG-sponge with (i) 2.0 mAh cm^−2^, (ii) 5.0 mAh cm^−2^, and (iii) 8.0 mAh cm^−2^ Li deposition (current density: 1.0 mA cm^−2^). Reproduced with permission [68]. Copyright 2020, Elsevier. (c) Schematic illustration and (d) cross-section SEM image of the CG host. (e) Voltage profiles of the symmetric cells at 5.0 mA cm^−2^ with a Li capacity of 1.0 mAh cm^−2^. Reproduced with permission [59]. Copyright 2020, Wiley-VCH. (f) Schematic illustration of the LNO-CGH host. (g) Schematic illustration and (h) cycling performance of the flexible quasi-solid LMBs. Reproduced with permission [69]. Copyright 2022, Elsevier.

As a highly compatible process for industrial fabrication, sputtering is considered to be a very promising approach for creating free-standing hosts. For instance, Li et al. constructed a conductive-dielectric gradient framework (CDG-sponge) by magnetron sputtering of a Ni nanolayer on the melamine sponge, where the structure of the gradient can be simply controlled by adjusting the sputtering time (Figure 2(a)) [68]. The exposed melamine with polar functional groups at the top functions as dielectric for distributing Li^+^ flux, while the electrically conductive bottom part acts as the platform for uniform Li nucleation and deposition. Such a gradient structure enables stable Li deposition/stripping behaviors and the upward growth of Li in the CDG-sponge. Cross-section scanning electron microscopy (SEM) images suggest that CDG-sponge exhibits a constant bottom-up Li metal deposition pattern without Li dendrite formation upon increasing Li plating capacity from 2.0 to 8.0 mAh cm^−2^ (Figure 2(b)). Compared with sputtering, vacuum filtration is more effective to build gradient structures due to its facile and low-cost nature. Hong and co-workers [59] presented a conductivity gradient heterofibrous scaffold (CG) based on a vacuum-assisted filtration process. The host consisted of a high conductivity Cu nanowires (CuNWs) bottom layer, an insulating cellulose nanofibers/SiO_2_ nanoparticles top layer, and a well-tailored conductivity CuNWs/cellulose nanofibers intermediate layer (Figures 2(c) and 2(d)). Both of their experimental and simulation results suggest that the top layer can sequester electrons, causing fast and appropriate Li^+^ fluxes in the bottom and middle layers, respectively. The unique structure successfully suppresses Li dendrite growth and renders a stable cyclic performance of 100 cycles under a high rate of 5.0 mA cm^−2^ (Figure 2(e)). Impressively, the full-cell assembled with LiNi_0.8_Co_0.1_Mn_0.1_O_2_ cathode exhibits 90.0% capacity retention with a high coulombic efficiency (CE) of 99.8% after 100 cycles. Recently, a topological host comprising gradient distributed SiC whiskers on carbon cloth (SiC/CC) was prepared by means of a gas-solid reaction between gaseous SiO and carbon nanofibers (CNFs) [57]. Thanks to the gradient distribution of SiC whiskers, the interwoven nanofibers form a topological structure, which confers conductivity gradient. With this design, SiC whiskers prevent electrons accumulation on the surface of SiC/CC host and simultaneously reduce the electric field intensity on its top region. Meanwhile, the porous structure with strong Li^+^ affinity reduces the local current density of the host and guarantees a relatively uniform Li^+^ flux in the pores. Accordingly, the full-cell assembled with Li@SiC/CC anode and LiFePO_4_ (LFP) cathode displays a capacity of 3.0 mAh cm^−2^ associated with a high average CE of 98.3% after 100 cycles at 0.2 C. Given that high specific surface area will aggravate the consumptions of electrolyte and Li, the special design of host is necessary [96, 97]. To reduce the interfacial side reactions, Zhou and co-workers [69] prepared a LiNO_3_-modified conductivity gradient host (LNO-CGH), which consists of a LiNO_3_ containing dielectric top layer and the bottom CNFs layers with upward decreased conductivity (Figure 2(f)). The steady release of LiNO_3_ from the dielectric top layer forms a firm nitride-rich SEI layer, leading to improved interfacial and thermodynamic stabilities of Li anode. While the conductivity gradient shifts the preferential Li deposition sites from the interface of anode and separator to the anode bottom, consequently, a dendrite-free deposition manner of the LNO-CGH host. Specifically, LNO-CGH with an upward Li growth process shows a steady CE of 97.3% over 200 cycles at 1.0 mAh cm^−2^ and 1.0 mA cm^−2^. Most impressively, flexible quasi-solid-state LMBs comprising the LNO-CGH@Li anodes, gel electrolytes, and flexible LiNi_0.8_Co_0.1_Mn_0.1_O_2_ cathodes manifest an initial capacity of ~ 191.0 mAh g^−1^ at 1 C and over 78.0% capacity retention even after 100 cycles (Figures 2(g) and 2(h)).

**Figure 3:**
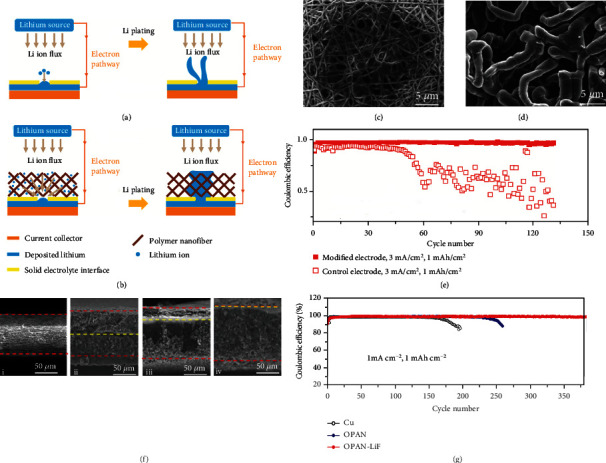
Schematic diagrams of the Li plating on the (a) bare Cu and (b) Cu-OPAN electrodes. SEM images showing smooth and mossy Li deposition pattern on the (c) Cu-OPAN and (d) bare Cu electrodes, respectively. (e) CE comparison of bare Cu and Cu-OPAN electrodes cycled at 1.0 mAh cm^−2^ and 3.0 mA cm^−2^. Reproduced with permission [70]. Copyright 2015, American Chemical Society. (f) Cross-section SEM images of the OPAN-LiF electrode (i) without and with (ii) 6.0 and (iii) 10.0 and (iv) 14.0 mAh cm^−2^ Li plating. (g) CE comparison of the electrodes at 1.0 mAh cm^−2^ and 1.0 mA cm^−2^. Reproduced with permission [72]. Copyright 2021, Elsevier.

Regarding the design of integrated hosts, Cui's group [70] first proposed an interesting electrode by placing an oxidized polyacrylonitrile (OPAN) network on the Cu current collector (Cu-OPAN). On one hand, the polar functional groups of OPAN prevent Li^+^ from concentrating around the “hot spots”, affording a relatively uniform Li^+^ flux, as schematically illustrated in Figures 3(a) and 3(b). On the other hand, the good affinity between the functional groups and electrolyte guarantees superior electrolyte uptake and accessibility. Furthermore, owing to its insulating nature, OPAN acts as the framework for guiding and confining Li growth along the fibers, leading to a planar morphology within the network (Figure 3(c)). On the contrary, Li deposition onto the bare Cu substrate exhibits a drastically different morphology, where the overgrowth of Li filaments is observed (Figure 3(d)). Under a practical current density of 3.0 mA cm^−2^ at 1.0 mAh cm^−2^, the average CE of the battery reaches 97.4% over 120 cycles (Figure 3(e)). Subsequently, a honeycomb-like hierarchical nitrogen-doped framework (HHNF) electrode was obtained by annealing PAN-coated Cu foil [71]. In this case, the pores promote the even distribution of electrons, while the lithiophilic N dopants guide homogeneous Li nucleation and deposition, leading to an “inside-outside” Li deposition pattern without dendrite formation in HHNF. Considering electrospinning is a simple and versatile approach to fabricate light-weight polymer fiber frameworks, functional additives could be readily introduced to further optimize the Li deposition behavior. For instance, Zhou and co-workers [72] reported a modified network by incorporating LiF into the OPAN fibers (OPAN-LiF). The incorporated LiF nanoparticles with low barrier energy for surface diffusion of Li^+^ guide dense Li deposition to cover the fibers instead of to outcrop the surface, which suppresses the growth of Li dendrites to a great extent. As shown in Figure 3(f), the thickness of OPAN-LiF increases upon increasing Li plating capacity from 6.0 to 14.0 mAh cm^−2^, where the deposited Li is consistently restrained inside the OPAN-LiF host. Specifically, the half-cell shows an average CE of 98.7% over 380 cycles with 1 mAh cm^−2^ Li capacity at 1.0 mA cm^−2^ (Figure 3(g)). After pairing with LFP cathode, the corresponding full-cell exhibits excellent cycling stability in terms of 89.0% capacity retention even after 1600 cycles at 5.0 C. Similarly, Ag nanoparticles modified OPAN framework (OPAN-Ag) was reported since the introduction of Ag could effectively decrease the Li nucleation barrier [73]. During Li deposition, Li-Ag alloy is initially formed, resulting in a low nucleation barrier of 40.0 mV at 1.0 mA cm^−2^. Meanwhile, Li-Ag alloy acting as a Li^+^ conductor endows the host with flat and dense Li deposition.

Except for PAN, poly-melamine-formaldehyde (PMF) has also been reported for designing integrated hosts [74]. Due to the nonconductivity property of PMF, Li only deposits on the plated Li substrate where Li nucleation occurs. The strong interactions between the N-containing polar groups (i.e., amine and triazine) of PMF and Li^+^ stimulate Li-ion redistribution and, more importantly, reduce the ion concentration gradient due to preferential ion flux near dendritic tips. Taking these advantages, the Cu current collector covered by PMF shows enhanced CE as compared to its bare counterpart under different electrochemical parameters (current density and cycling capacity). Even at an ultrahigh current density of 10.0 mA cm^−2^, the PMF/Li electrode after 50 cycles can still afford a high CE of 94.7% with low voltage hysteresis and interfacial resistance. Besides polymer fibers, glass fiber (GF) cloths were also demonstrated to be very effective in realizing dendrite-free LMAs [75].

**Figure 4:**
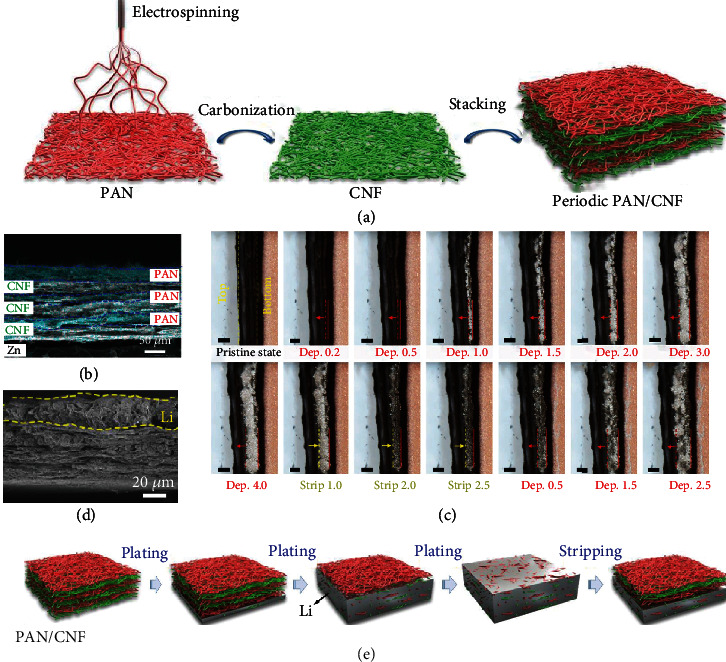
(a) Schematic illustration for fabricating the PAN/CNF host. (b) Zn elemental distribution mapping image of the host. (c) In situ optical photography observations of the electrolyte-electrode interface at 2.0 mA cm^−2^. (d) Cross-section SEM image of 20.0 mAh cm^−2^ Li deposited in the host. (e) Schematic illustrations showing Li plating and stripping within the PAN/CNF host. Reproduced with permission [76]. Copyright 2019, Wiley-VCH.

Despite controlled Li deposition in these gradient hosts, the undesired dendrites formed inside the skeleton during durable Li plating/stripping cannot be self-eliminated. To this end, Zou and co-workers [76] elaborately designed an interesting PAN/CNF host by periodically stacking conductive CNFs and dielectric PAN layers (Figure 4(a)). Zn-based nanoparticles were introduced into the lamellar structure to enhance its mechanical strength (Figure 4(b)). With this design, the electrode shows a bottom-up plating pattern, where Li preferentially deposits on the bottom conductive layer and then gradually fills the overhead layers (Figures 4(c)–4(e)). Even if uneven Li deposition occurs inside, the parasitic propagation will still be blocked by the overhead conductive layers in view of the fact that they are electrically equipotential for rehomogenizing the local electric field. This strategy enables a stable and smooth Li deposition, which is advantageous for high capacity cycling of up to 15.0 mAh cm^−2^. Furthermore, by recording the voltage fluctuations between the counter electrode and each conductive CNFs layer, the authors successfully monitored the real-time Li deposition process of CNF/polyimide (PI) [77].

## 3. Lithiophilicity Gradient

Considering that Li nucleation follows heterogenous nucleation model, the lithiophilicity of host can directly affect the eventual stability of the LMAs [98, 99]. Lithiophilicity is defined as the affinity of Li species to substrates [100], where Ag [101], Au [102], Zn [103], and ZnO [49] possessing strong binding energy with Li have so far demonstrated excellent lithiophilic property. Based on theoretical predictions and experimental verifications, we have recently correlated the structure of lithiophilic sites with Li electrochemistry, which reveals the lithiophilic sites dependency of Li deposition behavior in LMAs [104]. Generally, introducing lithiophilic components within host is beneficial to modulate its Li deposition behavior and the associated nucleation barrier [105]. However, with even distribution of lithiophilic substances in hosts, Li preferentially nucleates and deposits at their top zone, particularly under harsh conditions [106]. Constructing hosts with gradient distributed lithophilic materials has been demonstrated to be a proven method to inhibit Li dendrite growth. In this section, the latest progress for the lithiophilicity gradient designs of Li metal hosts will be summarized and discussed.

**Figure 5:**
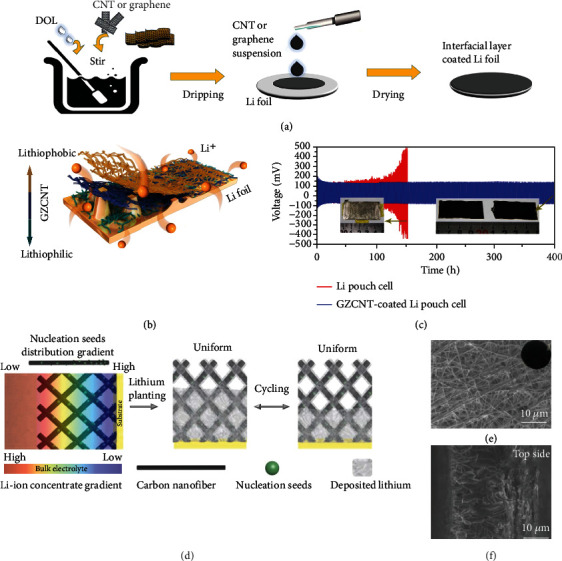
Schematic illustrations for (a) the fabrication of GZCNT and (b) Li deposition in GZCNT-coated Li foil. (c) Cycling performance of the symmetric pouch cells at 1.0 mA cm^−2^ and 1.0 mAh cm^−2^. The insets are corresponding photographs of Li (left) and GZCNT/Li foils (right) after 100 and 200 cycles, respectively. Reproduced with permission [78]. Copyright 2018, Springer Nature. (d) Schematic diagram of G-CNF in regulating Li deposition. (e) Top and (f) cross-section SEM images of the G-CNF/Li electrode with 5.0 mAh cm^−2^ Li deposition. The inset is the corresponding optical image. Reproduced with permission [79]. Copyright 2019, Wiley-VCH.

ZnO with good Li affinity is the most frequently used lithiophilic materials for fabricating lithiophilicity gradient hosts. As one of the earliest studies, Zhang and co-workers [78] designed a lithiophilicity gradient skeleton by dripping carbon nanotubes (CNTs) with different ZnO loadings layer by layer onto the Li foil (GZCNT) (Figure 5(a)). In such an architecture, the bottom layer composed of CNTs and lithiophilic ZnO is tightly fixed to Li foil, which affords a uniform SEI and prevents the intermediate mossy Li corrosion layer formation. While the robust lithiophobic CNTs top layer with porous structure plays an indispensable role in promoting Li^+^ diffusion and relieving the penetration of Li dendrites (Figure 5(b)), therefore, the symmetric cell of GZCNT/Li shows long-term stability at both high rate and high capacity, outperforming the bare Li and CNTs/Li references. Further symmetric pouch cells show over 200 cyclicity without visible dendrites detected on the electrode (Figure 5(c)). In addition, ~3.0 mAh cm^−2^ Li-S batteries are assembled, where the GZCNT/Li cells after 200 cycles present higher capacity retention (~58.0%) compared with their Li foil counterparts (~37.0%). Likewise, an electrospun CNFs host with gradient distributed ZnO particles (G-CNF) to modulate Li nucleation and deposition process was reported (Figure 5(d)) [79]. Benefiting from the accelerated Li deposition along the vertical direction, the electrode shows a dense and smooth deposition morphology. At 5.0 mAh cm^−2^ plating capacity, the electrode still shows dendrite-free surface owing to the bottom-growth mode of Li metal (Figures 5(e) and 5(f)). The symmetric cell presents a low hysteresis voltage of 8.0 mV even after 1700 h at 0.2 mA cm^−2^. To further improve the mechanical property of the matrix, graphene was introduced by a combination of modified multiple filtration and thermal curing techniques [80]. The presence of graphene not only leads to increased specific surface area for suppressing Li metal expansion, but also improved structural robustness for reducing protrusions at the electrode surface. Accordingly, the resulting gradient host (G-CZC) presents durable cycling with a low overpotential of about 15.0 mV for 920 h.

**Figure 6:**
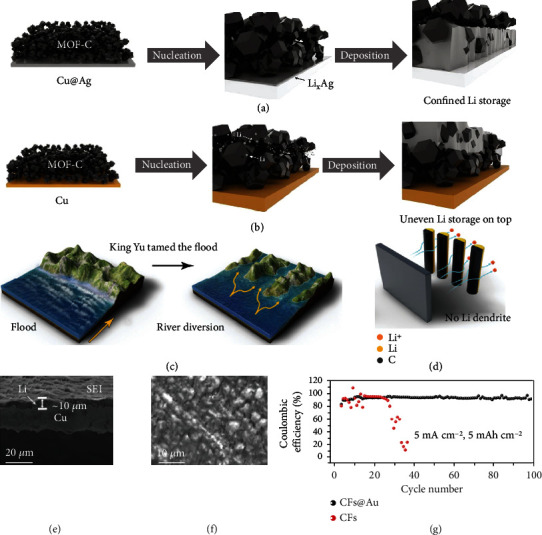
Schematic illustrations of Li deposition in (a) MOF-C/Cu@Ag and (b) MOF-C/Cu electrodes. Reproduced with permission [81]. Copyright 2022, Elsevier. Schematic diagrams of (c) “King Yu Tamed the Flood” and (d) Li deposition in the CFs@Au host. (e) Cross-section and (f) surface SEM images of Au nanoparticles modified Cu foil after 50 cycles and Li deposition, respectively. (g) CE of the electrodes cycled at 5.0 mA cm^−2^ and 5.0 mAh cm^−2^. Reproduced with permission [83]. Copyright 2018, Wiley-VCH.

Compared with ZnO, metals can directly alloy with Li without conversion reaction, hence no Li loss during predeposition [107]. Yun et al. [81] found that the insertion of Ag between MOF-derived carbon (MOF-C) layer and Cu substrate (MOF-C/Cu@Ag) could significantly lower the nucleation barrier of Li and, more importantly, enable a strong Li-substrate interaction for Li metal confinement. With the aids of in-operando synchrotron X-ray diffraction and SEM tests, they revealed that Ag can preferentially react with Li to form lithiophilic Li-Ag solid solution alloy during the early stage of Li plating, which induces Li deposition at the bottom of carbon layer. As deposition capacity increases, the Li-Ag alloy continuously optimizes Li growth within the carbon layer, thus effectively promoting pore utilization and Li metal confinement in MOF-C/Cu@Ag electrode (Figure 6(a)). In sharp contrast, without Ag coating, the MOF-C/Cu electrode shows preferential top Li deposition associated with pore clogging, resulting in insufficient pore utilization (Figure 6(b)). It is also noted that there is a trade-off between the kinetics of Li^+^ transport through the carbon layer and Li^+^ react with the substrate, which would provide a useful guidance for designing advanced carbon frameworks. Recently, a host of PVDF framework with gradient Ag nanoparticles decoration was reported by Zhao and co-workers [82] (C-Ag/PVDF). The electrical insulation at the top region restrains top Li growth, while the lithiophilic bottom region offers rich Li nucleation sites, leading to preferred and stable bottom-up deposition. Accordingly, the symmetric cell exhibits a long lifespan of 1300 h at 4.0 mA cm^−2^ and 4.0 mAh cm^−2^.

Similar to Ag, Au with high affinity to Li can also be used to construct lithiophilicity gradient hosts [36, 100, 106]. Motivated by the Chinese legend of “King Yu Tamed the Flood,” Xiang et al. [83] proposed a smart approach of combing the dredge and block to control Li^+^ diversion and deposit on the back side (Figure 6(c)). They selectively decorated the back side of CNFs film with Au nanoparticles (CFs@Au), which forces Li^+^ travel over its front surface and subsequently deposits from the opposite direction (Figure 6(d)). In this case, even if the formation of Li dendrites, they will always keep away from the separator, and hence no short-circuit risk. In addition, it is also found that a stable Au-containing SEI layer that is capable of suppressing the side reactions and accommodating volume variations of the electrode was detected, which contributes to the uniform and compact Li plating pattern in CFs@Au (Figures 6(e) and 6(f)). Remarkably, the Li||CFs@Au half-cell achieves a high CE of 98.0% after 100 cycles under a high rate of 5.0 mA cm^−2^ and capacity of 5.0 mAh cm^−2^ (Figure 6(g)). In addition to metal, nonmetallic Si is another good candidate to alloy with Li. As reported by Yan and co-workers [85], the host with gradually decreased Si content from bottom to top (denoted as GSCP) can not only guide preferential Li nucleation and growth in a bottom-up way but also guarantee high space utilization. At a high rate of 10.0 C, the full-cell assembled with Li_4_Ti_5_O_12_ cathode still displays 84.5% capacity retention after 5000 cycles associated with a high average CE of 99.98%. It is worth mentioning that the asymmetric bottom deposition will cause tensile stress in the upper part, leading to the bend or even failure of the electrode during long-term operation. In this regard, Cheng et al. [86] developed an interesting host (CuCNF-NCNF) comprising a gradient nitrogen-doped CNFs layer (NCNF) covered on a Cu-doped CNFs layer (CuCNF). The NCNF with abundant nucleation sites guides smooth Li deposition, whereas the CuCNF with high mechanical strength acts as “reinforcing rib” to resist the fatigue stress during cycling. Such a unique structure endows the full-cell with a high capacity of 100.2 mAh g^−1^ even at a high rate of 5.0 C. Different from the above discussed gradient structure, the host with reversely designed architecture, meaning higher Li affinity in the top region, has also been proved to be an effective pathway to suppress Li dendrite growth. Typically, Zhang and co-workers [87] reported a CuNW current collector with gradually decreased lithiophilic Cu_3_P along the cross-section, where the gradient was formed via a phosphidation-controlled process. Upon deposition, the reaction between top Cu_3_P and Li^+^ generates a Li_3_P-rich surface, which homogenizes the Li^+^ flux on the electrode. In addition, the less phosphidized bottom part maintains a high conductivity to ensure good electron transfer. Consequently, the electrode presents steady Li nucleation and deposition, and a high Li mass loading of 44 wt% is achieved. The contradictory result leads to an ambiguous mechanism of lithiophilicity gradient design in regulating Li deposition, leaving a large room for further studies.

## 4. Dual Gradient

**Figure 7:**
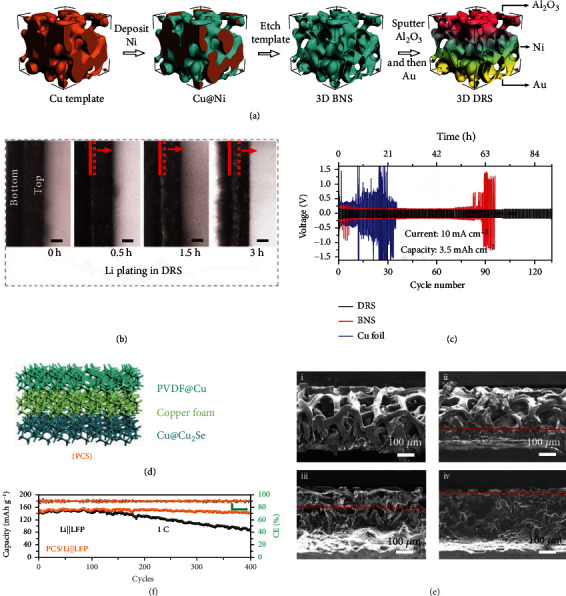
(a) The fabrication process of DRS. (b) Optical microscopy images of the DRS during Li plating. (c) Voltage profiles of the electrodes at 3.5 mAh cm^−2^ and 10.0 mAcm^−2^. Reproduced with permission [88]. Copyright 2019, Springer Nature. (d) Schematic illustration of the PCS electrode. (e) Cross-section SEM images of the PCS electrode (i) at its original state and with (ii) 2.0, (iii) 5.0, and (iv) 20.0 mAh cm^−2^ Li plating, respectively. (f) Cycling performances of the Li||LFP and PCS/Li||LFP cells at 1.0 C. Reproduced with permission [91]. Copyright 2022, American Chemical Society.

Dual-gradient host combines the advantages of conductivity gradient and lithiophilicity gradient, which synergistically addresses the issue of uncontrolled Li growth [108]. Typically, Pu et al. [88] obtained a deposition-regulating scaffold (DRS) by coating Al_2_O_3_ and Au at the top and bottom regions of bare nickel scaffold, respectively (Figure 7(a)). The top region with Al_2_O_3_ coating presents lower conductivity as compared to the bottom with Au, forming a conductivity gradient. Meanwhile, lithiophilicity gradient is simultaneously created due to the higher Li affinity of Au than Al_2_O_3_. Owing to the shift of favorable Li nucleation sites from the anode/separator interface to the DRS bottom (Figure 7(b)), the DRS electrode displays a high CE of ~ 98.1% over 500 cycles at 3.5 mAh cm^−2^ and 2.0 mA cm^−2^. Meanwhile, the symmetric cells show long-term cycling over 120 cycles at 10.0 mA cm^−2^ (Figure 7(c)). Impressively, the cells maintain excellent stability no matter under extreme electrochemical (40.0 mAh cm^−2^) or environmental (-15°C) conditions, suggesting the great prospect of DRS for LMBs. In another work, Yun and co-workers [89] came up with a Cu framework electrode, where Ag was used to activate its bottom associated with PVDF to passivate the top (PVDF/Cu/Ag). Since electrons and lithiophilic sites mainly concentrated at the bottom region, the electrode demonstrates bottom-up Li deposition without dendrites formation. Thereafter, a dual-gradient architecture (ZIF-8/C-ZIF-8) is constructed by adopting zeolitic imidazolateframework-8 (ZIF-8) as the surface passivation layer and carbonized ZIF-8 (C-ZIF-8) as the bottom electron/Li^+^ conductive layer, respectively [90]. After consecutively covering C-ZIF-8 and ZIF-8 on a Cu current collector, the resulting electrode delivers a longer cycling performance than that of bare Li. Likewise, Huang and co-workers [91] fabricated a gradient host of PVDF@Cu@Cu_2_Se (PCS) by coating Cu foam with two different layers (Figure 7(d)). The upper insulating PVDF layer efficiently impedes Li deposition at the anode/separator interface, while the lower Cu_2_Se layer guides Li^+^ preferentially to deposit at the bottom region (Figure 7(e)). Additionally, Cu_2_Se can also partially react with Li^+^ to form highly ion-conductive Li_2_Se, which acts as the artificial SEI to facilitate rapid Li^+^ transportation. As a consequence, the PCS/Li||LFP full-cell shows a capacity retention of 94.4% at 1 C for 450 cycles (Figure 7(f)), much higher than that of Li||LFP (53.5%).

Unlike PAN, the blend of PAN and poly (vinylidene fluoride-co-hexafluoropropylene) (PVDF-HFP) can acquire a trade-off between the affinity to Li^+^ and adaptivity of conformal coating on substrate. As reported by Zhang et al. [92], a poly/Cu mesh/Au host constructed by successively coating Au nanoparticles and PVDF-HFP on the top and bottom of Cu mesh facilitates long-term cycling stability of the electrode. The full-cell assembled with LFP cathode presents stable cycling for 150 cycles (current rate: 2.0 C, capacity retention: 96.4%) and good rate capacity (100.3 mAh g^−1^ at 3.0 C). Recently, Zheng and co-workers [93] prepared a unique porous Cu-Au-ZnO-PAN-ZnO host (CAZPZ) through the combination of electrostatic spinning and magnetron sputtering. The high conductivity and lithiophilicity region composed of Au and ZnO not only provides abundant Li nucleation sites but also decreases the Li nucleation overpotential. On the other hand, the upper of ZnO-PAN-ZnO skeleton with poor conductivity renders enough space to accommodate Li. Furthermore, the reaction between ZnO and Li^+^ forms a highly ion-conductive and lithiophilic Li_2_O/Li_x_Zn layer during initial Li plating, guaranteeing uniform Li^+^ flux and high mechanical strength. Benefiting from the novel design, the CAZPZ/Li||LFP full-cell delivers good cyclicity for 1000 cycles at 5.0 C with 97.3% capacity retention. Following the same concept, the authors further synthesized a Si@CNFs@ZnO skeleton-coated ZnO-Cu foil (denoted as SCZ) dual-gradient host to avoid the top-growth of Li metal [94].

## 5. Conclusion and Perspective

In order to solve the issues of LMAs, considerable efforts so far have been devoted to developing novel hosts with gradient structures of lithiophilicity and/or conductivity. According to their structures and associated mechanisms for Li deposition, gradient hosts can be divided into three categories including lithiophilicity gradient host, conductivity gradient host, and lithiophilicity-conductivity dual-gradient host. For the former two architectures, they present gradually decreased conductivity or lithiophilicity from their bottom to the top surface. As a result, more electrons or Li ions are accumulated at the bottom region of the hosts, where Li deposition preferentially occurs. However, undesirable top Li deposition is still noted under the harsh conditions of high capacity and/or high rate. In lithiophilicity-conductivity dual-gradient structure, the bottom of the hosts is both more conductive and lithiophilic than their top zone, consequently more favorable Li nucleation and deposition. The simultaneous regulation of electrons and Li ions in the hosts leads to a synergistic effect in inhibiting the uncontrollable formation of Li dendrites. In this review, the recent progress in the gradient designs of Li metal hosts for dendrite-free LMBs is discussed (Table 1). Such well-constructed hosts not only effectively regulate Li nucleation and deposition but also dramatically alleviate the volume swings during cycling, thus realizing the suppression of Li dendrites and remarkable improvement of cyclicity.

Despite this encouraging progress, gradient design of hosts is still in infancy and requires more in-depth studies. The directions of future research are summarized as follows:

1. Characterization techniques and theoretical simulations. Li deposition is an extremely complex process, which is closely related to the properties of host and electrolyte, as well as other factors (such as current density and SEI). Given that Li metal and SEI are highly sensitive to moisture and air, advanced analytical techniques, such as cryo-electron microscopy and in situ characterizations, are expected to observe the dynamic Li plating/stripping processes in a realistic battery. Moreover, the quantitative understanding of the deposition process is still challenging. Further theoretical simulations, for example, finite element modeling, are expected to provide a deeper understanding of the evolution of Li plating/stripping in host materials [109–113].
2. Porosity contributions and electrode thickness design. In addition to conductivity and lithiophilicity, the pore structures (pore diameter, pore depth, and pore tortuosity) and the thickness of composite anode can also affect the distribution of electric and Li^+^ concentration fields during Li plating/stripping. Therefore, the contributions of porosity for smooth Li deposition need to be clarified. Also, the electrode thickness should be rationally designed and clearly indicated.
3. Failure mechanism. It is well known that the introduction of lithophilic sites can reduce the Li nucleation barrier of host. However, these lithiophilic active sites may be covered by dead Li upon cycling and thus no longer work on guiding Li deposition [114]. Therefore, the failure mechanism of lithiophilic sites needs to be investigated.
4. Practical application. Since host is an inactive component of battery, its content should be reduced as much as possible so that to achieve over 400 Wh Kg^−1^ LMBs. Specifically, the mass ratio of host in the composite host should be ≤50 wt%. Moreover, the electrochemical performances of currently reported gradient hosts are mostly analyzed by coin cells, which cannot provide conclusive information for further performance improvement. Pouch cells assembled with N/P ratio <3.0, electrolyte content <3.0 g Ah^−1^, working current density >3.0 mA cm^−2^, and areal capacity >3.0 mAh cm^−2^ are highly essential for evaluating the practical application of the hosts.

## Figures and Tables

**Table 1:** Electrochemical performance comparison of the hosts with different gradient structures.

Samples	Half-cell (CE, current density, capacity, cycle) (mA cm^−2^/mAh cm^−2^)	Symmetric cells (cycle, current density, capacity) (h/mA cm^−2^/mAh cm^−2^)	Full-cell (capacity retention)	Capacity (mAh cm^−2^)	Electrolyte	Ref.
Conductivity gradient						
CDG-sponge	98.4%, 0.5, 1.0, 500	780, 1.0, 1.0	1.0 C, 99.4%, 100 cycles (cathode: LFP)	3.0	1 M LiTFSI in DOL&DME (1 : 1 in v/v) ^a^	[68]
CG	N.A.	500, 1.0, 1.0	1.0 C, 90.0%, 100 cycles (cathode: LiNi_0.8_Co_0.1_Mn_0.1_O_2_)	6.0	1 M LiPF_6_ in EC&DEC (1 : 1 in v/v) ^a,b^	[59]
SiC/CC	N.A., 5.0, 3.0, 50	1000, 1.0, 1.0	0.5 C, 80.0%, 120 cycles (cathode: LFP)	3.0	1 M LiTFSI in DOL&DME (1 : 1 in v/v) ^a^	[57]
LNO-CGH	97.3%, 1.0, 1.0, ~200	N.A.	1.0 C, 74.9%, 100 cycles (cathode: LiNi_1/3_Co_1/3_Mn_1/3_O_2_)	5.0	1 M LiPF_6_ in EC&DEC (1 : 1 in v/v) ^b^	[69]
Cu-OPAN	97.4%, 3.0, 1.0, 120	>80, 3.0, 1.0	N.A.	N.A.	1 M LiTFSI in DOL&DME (1 : 1 in v/v) ^a^	[70]
HHNF	98.5%, 0.5, 1.0, 400	1000, 0.5, 1.0	0.5 C, N.A., 150 cycles (cathode: LFP)	3.0	1 M LiPF_6_ in EC&DEC (1 : 1 in v/v)	[71]
OPAN-LiF	97.5%, 3.0, 1.0, 180	N.A.	5.0 C, 89.0%, 1600 cycles (cathode: LFP)	2.5	1 M LiTFSI in DOL&DME (1 : 1 in v/v)	[72]
OPAN-Ag	95.6%, 1.0, 1.0, 125	>1000, 0.5, 1.0	0.5 C, 84.0%, 100 cycles (cathode: LiNi_0.9_Co_0.1_O_2_)	4.0	1 M LiPF_6_ in EC&DEC (1 : 1 in v/v) ^b^	[73]
PMF	N.A., 1.0, 1.0, 400	350, 2.0, 1.0	2.0 C, N.A., 1000 cycles (cathode: Li_4_Ti_5_O_12_)	20.0	1 M LiPF_6_ in EC&DEC (1 : 1 in v/v)	[74]
GF	97.0%, 1.0, 0.5, N.A.	N.A.	N.A.	N.A.	1 M LiTFSI in DOL&DME (1 : 1 in v/v) ^a^	[75]
PAN/CNF	N.A., 1.0, 5.0, >100	1800, 1.0, 1.0	1.0 C, 70.0%, 100 cycles (cathode: LiNi_0.8_Mn_0.1_Co_0.1_O_2_)	10.0	1 M LiPF_6_ in EC&DEC&DMC (1 : 1 : 1 in v/v)	[76]
CNF/PI	97.5%, 1.0, 3.0, 140	N.A.	1.0 C, 74.4%, 100 cycles (cathode: LiNi_0.8_Mn_0.1_Co_0.1_O_2_)	5.0	1 M LiPF_6_ in EC&DEC&DMC (1 : 1 : 1 in v/v) ^b^	[77]
Lithiophilicity gradient						
GZCNT	N.A.	1000, 1.0, 1.0	0.2 C, N.A., 200 cycles (cathode: S)	N.A.	0.6 M LiTFSI in DOL&DME (1 : 1 in v/v) ^a^	[78]
G-CNF	98.1%, 0.5, 0.5, 700	1700, 0.2, 0.2	1.0 C, 95.7%, 300 cycles (cathode: LFP)	5.0	1 M LiPF_6_ in EC&DMC&EMC (1 : 1 : 1 in v/v) ^d^	[79]
G-ZGC	~98.2%, 1.0, 1.0, 500	920, 1.0, 1.0	N.A.	5.0	1 M LiPF_6_ in EC&DEC (1 : 1 in v/v)	[80]
MOF-C/Cu@Ag	N.A.	500, 0.4, 0.4	N.A.	N.A.	1 M LiTFSI in DOL&DME (1 : 1 in v/v) ^a^	[81]
C-Ag/PVDF	>96.0%, 0.5, 1.0, 180	>1300, 4.0, 4.0	0.5 C, 101.7%, 200 cycles (cathode: LFP)	3.0	1 M LiPF_6_ in EC&DEC (1 : 1 in v/v)	[82]
CFs@Au	99.2%, 1.0, 1.0, 400	>700, 1.0, 2.0	0.1 C, N.A., 100 cycles (cathode: S)	2.4	1 M LiTFSI in DOL&DME (1 : 1 in v/v) ^a^	[83]
Au/CP	97.6%, 2.0, 1.0, 100	N.A.	0.5 C, N.A., 500 cycles (cathode: LFP)	N.A.	1 M LiPF_6_ in EC&DEC&DMC (1 : 1 : 1 in v/v) ^c^	[84]
GSCP	99.0%, 1.0, 1.0, 400	>1350, 1.0, 1.0	10.0 C, 84.5%, 5000 cycles (cathode: Li_4_Ti_5_O_12_)	3.0	1 M LiTFSI in DOL&DME (1 : 1 in v/v) ^a^	[85]
CuCNF-NCNF	~96.0%, 2.0, 1.0, 250	1000, 1.0, 1.0	0.5 C, 95.8%, 500 cycles (cathode: LFP)	2.0	1 M LiPF_6_ in EC&DEC&DMC (1 : 1 : 1 in v/v)	[86]
CuNW-P	97.4%, 1.0, 1.0, 150	1000, 1.0, 1.0	0.5 C, N.A., 300 cycles (cathode: LFP)	3.0	1 M LiTFSI in DOL&DME (1 : 1 in v/v) ^a^	[87]
Dual gradient						
DRS	98.1%, 1.0, 1.0, 500	500, 2.0, 3.5	N.A.	N.A.	1 M LiTFSI in DOL&DME (1 : 1 in v/v) ^a^	[88]
PVDF/Cu/Ag	>98.0%, 0.5, 1.0, >200	250, 1.0, 1.0	N.A.	5.0	1 M LiTFSI in DOL&DME (1 : 1 in v/v) ^a^	[89]
ZIF-8/C-ZIF-8	97.6%, 1.0, 1.0, >300	>700, 1.0, 1.0	2.0 C, 56.0%, 160 cycles (cathode: LiCoO_2_)	3.0	1 M LiPF_6_ in EC&DEC (1 : 1 in v/v) ^b^	[90]
PCS	97.2%, 2.0, 1.0, 150	>1000, 1.0, 1.0	1.0 C, 94.4%, 450 cycles (cathode: LFP)	2.0	1 M LiTFSI in DOL&DME (1 : 1 in v/v) ^a^	[91]
Poly/Cu mesh/Au	>96.1%, 0.5, 2.0, >200	400, 1.0, 1.0	2.0 C, 96.4%, 150 cycles (cathode: LFP)	3.0	1 M LiPF_6_ in EC&DMC (1 : 1 in v/v)	[92]
CAZPZ	99.0%, 0.5, 1.0, N.A.	200, 3.0, 1.0	5.0 C, 97.3%, 1000 cycles (cathode: LFP)	5.0	1 M LiTFSI in DOL&DME (1 : 1 in v/v) ^a^	[93]
SCZ	97.5%, 1.0, 1.0, 210	>900, 1.0, 1.0	5.0 C, 94.8%, 2000 cycles (cathode: LFP)	5.0	1 M LiTFSI in DOL&DME (1 : 1 in v/v) ^a^	[94]

(1) Electrolyte with (a) LiNO_3_, (b) FEC, (c) VC, and (d) EC as the additives, respectively. (2) 1 C value: LiNi_0.9_Co_0.1_O_2_ = 200 mA g^−1^, LiNi_0.8_Mn_0.1_Co_0.1_O_2_ = 200 mA g^−1^, LiNi_1/3_Co_1/3_Mn_1/3_O_2_ = 150 mA g^−1^, LFP = 170 mA g^−1^, S = 1675 mA g^−1^, Li_4_Ti_5_O_12_ = 175 mA g^−1^, LiCoO_2_ = 274 mA g^−1^.
